# Effect of Porosity on Tribological Properties of Medical-Grade 316L Stainless Steel Manufactured by Laser-Based Powder Bed Fusion

**DOI:** 10.3390/ma18030568

**Published:** 2025-01-26

**Authors:** Germán Omar Barrionuevo, Magdalena Walczak, Patricio Mendez, Iván La Fé-Perdomo, Erika Chiluisa-Palomo, Wilson Navas-Pinto, Duncan E. Cree

**Affiliations:** 1 Departamento de Ciencias de la Energía y Mecánica, Universidad de las Fuerzas Armadas ESPE, Sangolquí 171103, Ecuador; 2 Department of Engineering, Universidad Católica del Uruguay, Av. 8 de octubre 2738, Montevideo 11600, Uruguay; 3 Department of Mechanical and Metallurgical Engineering, School of Engineering, Pontificia Universidad Católica de Chile, Av. Vicuña Mackenna 4860, Santiago 7820436, Chile; 4 Chemical and Materials Engineering Department, University of Alberta, Edmonton, AB T6G 2R3, Canada; 5 Escuela de Ingeniería Mecánica, Pontificia Universidad Católica de Valparaíso, Valparaíso 2340025, Chile; 6 Facultad de Ciencias de la Ingeniería y Aplicadas, Universidad Técnica de Cotopaxi, Latacunga 050101, Ecuador; 7 Department of Mechanical Engineering, McMaster University, Hamilton, ON L8S 4L7, Canada

**Keywords:** laser powder bed fusion, porosity, wear, tribology, biomedical materials, 316L stainless steel

## Abstract

The potential of laser-based powder bed fusion (L-PBF) technology for producing functional components relies on its capability of maintaining or even improving the mechanical properties of the processed material. This improvement is associated with the microstructure resulting from the high thermal gradient and fast cooling rate. However, this microstructural advantage may be counterbalanced by the lack of full density, which could be tolerated to a certain degree for applications such as biomedical implants and medical equipment. In this study, medical-grade 316L stainless steel specimens with porosities ranging from 1.7 to 9.1% were additively manufactured by L-PBF using different combinations of laser power and scanning speeds. Tribological properties were evaluated by pin-on-disc testing in dry conditions against a silicon nitride test body and analyzed in the context of microstructural characterization by optical and electron microscopy. The results reveal that higher porosity allows for a diminishing wear rate, which is explained by the capacity of the pores to retain wear debris related with the three-body abrasion. This research provides practical insights into the design of medical wear-resistant components, thereby enhancing our understanding of the potential of L-PBF in the fields of materials science and biomedical engineering.

## 1. Introduction

The laser-based powder bed fusion (L-PBF) technology relies on building 3D objects one layer at a time, employing a high-power laser (>100 W) to fuse a powder bed. Each consecutive layer bonds to the previous one via localized melting, which, with proper geometric control, results in the “growth” of a functional component [1,2,3]. However, additive manufacturing (AM) technologies are associated with inherent challenges, such as heterogeneity due to the anisotropy of the resulting mechanical properties, primarily hardness and elongation [4,5,6], and internal defects such as pores and non-metallic inclusions originating from the quality of the raw powder [7,8,9]. In addition, the processing parameters and the metal powder dispersion system influences the relative densities of additively manufactured parts [10,11].

As a rule of thumb, it has been proposed that components obtained by L-PBF must have a very high relative density (>99%) for them to be useful for conventional engineering applications such as aerospace components, automotive parts, implants, or prosthetics [12,13]. Nonetheless, materials of higher porosity have not been discarded due to their biomimetic potential [14], enabling the reduction in the elastic modulus, which is particularly relevant for medical implants [15]. In this context, porosity can be used for further functionalization of the material, taking advantage of the high surface area and the capacity for permeability, self-lubrication, and biocompatibility [15,16] for which controlling the size, distribution, and connectivity of the pores is essential [6,16,17]. In that sense, L-PBF technology is applicable for tuning porosity parameters by adjusting the processing protocol [16,17].

The specific advantages of porous materials can be tailored by controlling the size, distribution, and connectivity of the pores, allowing for new functions in engineering and biomedical applications [7,17,18]. Porosity levels are essential, but mechanical properties are dependent on porosity in terms of the number of pores (i.e., the fractional porosity), their interconnection, size, morphology, distribution, chemical composition, and lubricant availability. The interdependence of morphological characteristics and functional properties is of particular importance for tribological applications. On the one hand, the presence of pores generally reduces the overall material hardness, which allows for mitigating the stress shielding of medical implants [19,20,21] but can lead to increased wear rates [22] and decreased load-bearing capacity of the material, making it more susceptible to deformation and further material removal under load. On the other hand, porous materials can retain wear debris, preventing them from acting as an abrasive and thus reducing wear [23,24]. However, too high of a porosity can also compromise fracture toughness by providing sites for crack initiation and propagation.

Porous surfaces can also retain lubricants within their structure, potentially reducing friction by providing a consistent lubricant supply at the contact interface [25,26]. Moreover, these craters, or pores, can act as liquid reservoirs [27]. The size of surface craters can change the lubrication response. For example, larger crater sizes affect the deterioration of the properties of the liquid spreading over the mating surfaces. In that regard, research evaluating the tribological performance and wear mechanisms of additively manufactured steel parts remains open to discussion. While several researchers have studied the wear properties of L-PBF samples, their research has primarily focused on the process parameters of these properties [28,29,30].

Austenitic 316L stainless steel (SS) is suitable for L-PBF processing since it has a wide range of potential applications due to its excellent corrosion resistance, biocompatibility, recyclability, and has gained experience in engineering and biomedical applications [31,32]. Kumar [33] examined the effects of chemical composition and microstructure on wear properties of an iron-based selective laser sintering (SLS) material and determined that an increase in iron content led to a rise in the body centered cubic (BCC) phase and a decline in wear properties. For the specific case of 316L SS, Barrionuevo et al. [34] compared the wear resistance of additively manufactured (L-PBF) 316L SS against a conventionally processed material. The results showed there was a 30% higher wear resistance in the case of the L-PBF samples. This suggests additively manufactured materials could offer potential for applications requiring increased wear resistance.

Several authors have evaluated the performance of 316L SS in dry [35,36] and lubricated [37,38] conditions. Mandev et al. [36] reported that 316L SS alloy was suitable for thermal applications due to its extended surface application and transpiration cooling, while Chen et al. [39] explained that the wear mechanisms of SS depends not only on the applied loads, but, to a greater extent, on the microstructure. In this sense, applying heat treatments is not recommended to improve its wear resistance [40].

Therefore, investigating the effect of porosity on the tribological response of additively manufactured biomaterials is necessary to further the process-controlled porosity in an effort to customize wear resistance. The present study undertakes a systematic approach for relating the laser power and scanning speed during the additive manufacturing of L-PBF 316L SS. The wear resistance is evaluated by taking into account the porosity of the materials. In particular, it is hypothesized that tuning of tribological properties is possible without compromising mechanical strength.

## 2. Materials and Methods

### 2.1. Sample Preparation by L-PBF

**Figure 1:**
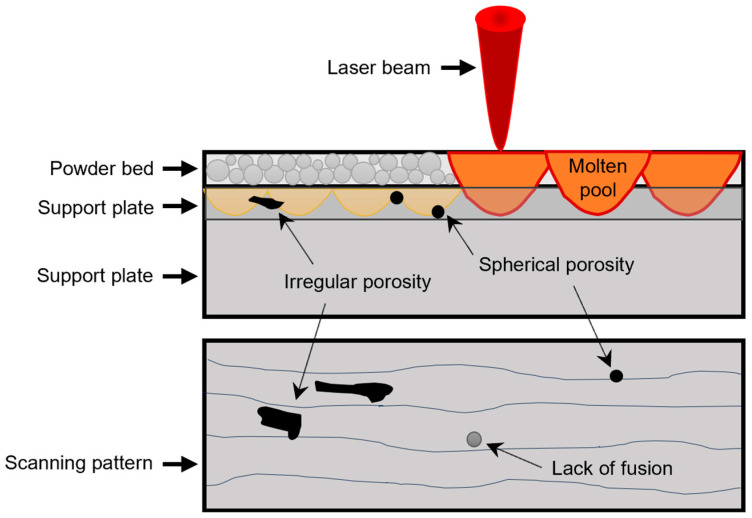
Schematic representation of the laser powder bed fusion process where the different types of porosity generated can be observed: section view (**top**) and top view (**bottom**).

Austenitic stainless steel of medical grade AISI 316L was used to fabricate prismatic samples with dimensions of 16 × 11 × 6 mm^3^ (Figure 1). The prime material was a metallic powder with an average particle size of around 33 µm (ranging from 18 to 49 µm) with a nominal chemical composition in wt% as follows: 16.5–18.5% Cr, 10–13% Ni, 2–2.5% Mo, ≤2% Mn, ≤1% Si, ≤0.45% P, ≤0.3% C, ≤0.3% S, and Fe (balance).

A Concept Laser (MLAB 200R) L-PBF system (Lichtenfels, Germany) was employed to manufacture the samples additively. The setup featured a 200 W (Nb: YAG) fiber laser, wavelength 1064 nm. A scanning strategy featuring 67° inter-layer rotation was adopted, and a full-factorial design of experiments of two factors (laser power and scanning speed) at three levels was utilized (Table 1). These parameters were chosen to assure reasonable results based on preliminary runs. Hatch spacing of 60 µm and a layer thickness of 30 µm were kept constant to reduce the number of experimental runs. Nitrogen was used as a shielding agent to prevent the molten metal from reacting with oxygen.

### 2.2. Microstructural Characterization

The porosity of the samples was determined using Archimedes’ principle as the complement of the relative density (*RD*), shown in Equations (1) and (2). Three replicas of each experimental run were evaluated to certify the reliability of the results.

(1) $$RD=\left(\frac{m_{a}}{m_{a}-m_{w}}\right)\left(\frac{\rho_{w}}{\rho_{T}}\right)$$

(2) $$Porosity\left(\%\right)=1-RD$$

where $m_{a}$ is the mass of the sample in the air, $m_{w}$ is the mass of the distilled water, $\rho_{w}$ is the density of the distilled water, and $\rho_{T}$ is the theoretical density of the 316L SS (7.9 g/cm^3^). The morphology of the external surface was inspected by optical microscopy (OM) (Nikon Eclipse MA200, Tokyo, Japan) and by scanning electron microscopy (SEM) equipped with an energy-dispersive spectroscopy (EDS) analyzer (TESCAN—MIRA 3, Brno, Czech Republic). The OM and SEM micrographs were processed and analyzed using Fiji software (2.16.0) to calculate the porosity level through the threshold tool [41].

The shape factor (SP) calculated by Equation (3) was employed to gain information on the pore morphology. Per represents the perimeter of the pores, and A is the pore area.

(3) $$SP=\frac{{Per}^{2}}{4\pi A}$$

The experimental results were analyzed using Minitab 19^®^ to determine the statistical effect of the processing parameters.

### 2.3. Tribological Characterization

A pin-on-disc tribometer (CSM Instruments, Delémont, Switzerland) evaluated the sliding wear resistance in dry conditions. The tests were conducted with a normal force of 5 N at a sliding speed of 0.0167 m s^−1^, covering a sliding distance of 30 m with a track diameter of 2 mm. The head of the pin was a silicon nitride (Si_3_N_4_) sphere with a pin-end diameter of 5 mm and an average hardness of 1550 HV, which was used as the counterbody.

Volume loss and the wear track were determined using a 3D optical profiler (Zygo, ZeGage, Middlefield, CT, USA). Each of the reported results was an average of over three measurements. In addition, the coefficient of friction (µ) was obtained during the sliding wear test, and the specific wear rate (ω) was calculated by Equation (4):

(4) $$\omega =\frac{V_{l}}{F\times d}$$

where *ω* is measured in [mm^3^ N^−1^m^−1^], $V_{l}$ is the volume loss in [mm^3^], *F* is the normal force [N], and *d* is the sliding distance [mm].

### 2.4. Characterization of Nanomechanical Properties

Nanoindentation tests were performed on a porosity-free site with a normal force of 150 mN and a holding time of 10 s using a nanoindentation tester (NHT3) equipped with a standard Berkovich indenter. The Oliver and Pharr method [42] was employed to determine the nanohardness (*H*) and the reduced elastic modulus (*Er*) from the loading-unloading curve. The value of *H* was determined by applying Equation (5):

(5) $$H=\frac{F_{max}}{A_{c}}=\frac{F_{max}}{26.43\;{h_{c}}^{2}\;}$$

where $F_{max}$ is the maximum indentation force [mN], and *h_c_* is the contact depth under the maximum force [nm]. The reduced elastic modulus was evaluated by Equation (6):

(6) $$\frac{1}{E_{r}}=\frac{1-{v_{s}}^{2}}{E_{s}}+\frac{1-{v_{i}}^{2}}{E_{i}}$$

where, $v_{s}$ and $v_{i}$ are the Poisson ratio of the sample and the indenter, respectively, and $E_{s}$ and $E_{i}$ are the Young moduli [GPa] of the samples and the indenter, respectively.

## 3. Results

### 3.1. Porosity

**Figure 2:**
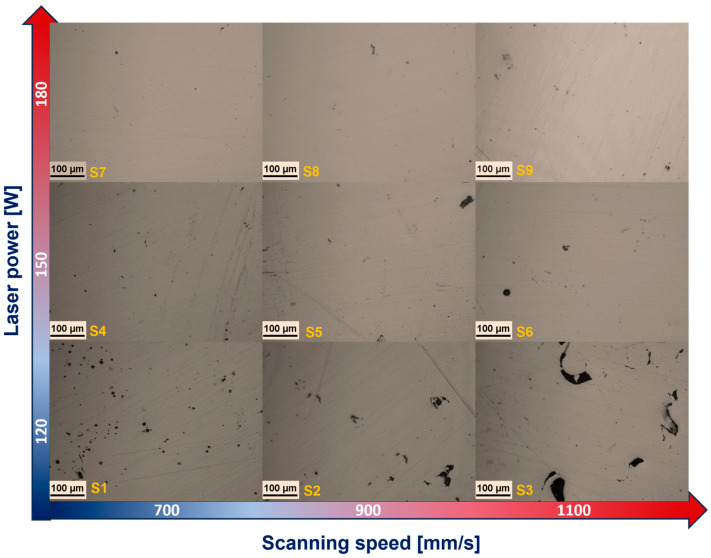
OM images showing the porosity levels of the 316L stainless steel processed by L-PBF using varying scanning speeds and laser power.

**Figure 3:**
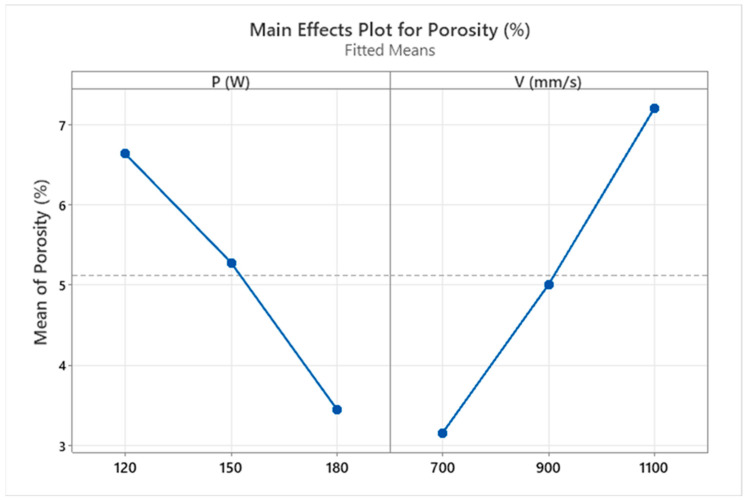
Main effect plot of the laser power (P) and scanning speed (V) on the resulting mean porosity.

Figure 2 shows the effect of varying the laser power and the scanning speed on the resulting porosity. At a high laser power (180 W), the material presented low porosity, while the speed had an inverse effect. For example, at a scanning speed of 700 mm/s, there was a lower level of porosity compared to a speed of 1100 mm/s where the porosity increased. As reported by several authors [10,26,43,44], insufficient laser energy density tends to produce non-melted and voided regions in the powder layer. This can be statistically verified in the main effect plot graph for porosity (Figure 3).

An average porosity of 5% was obtained when the samples were manufactured with an average laser power (P) and scanning speed (V). To reduce the porosity level, it is recommended to reduce the V and increase the P, while to increase the porosity, it is suggested to increase the V and reduce the P. Table 2 reports the average porosity results with their standard deviations. Using Archimedes’ principle, the sample with the highest porosity (9.1%) was obtained using a low power and high scanning speed. In comparison, the sample with the lowest porosity (1.7%) was obtained with a higher power and lower speed. The porosity calculated by image correlation was lower (<3%). This difference is because the surface porosity did not consider the existing pores inside the samples.

**Figure 4:**
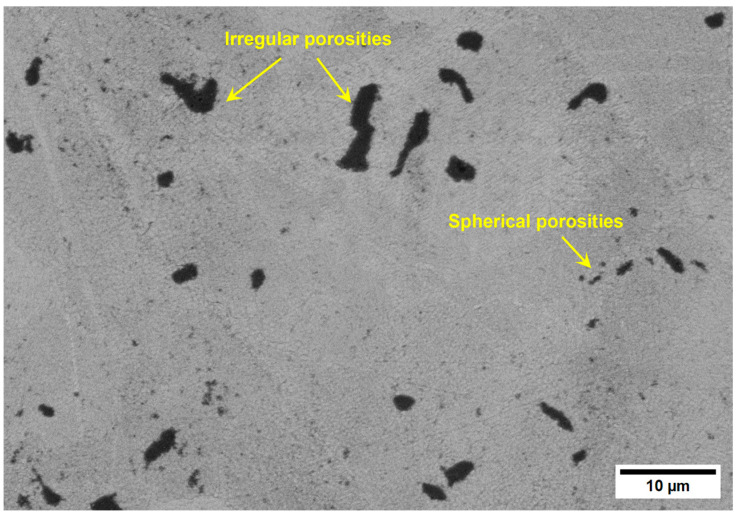
SEM image showing the pore morphology and distribution of the sample S3.

Figure 4 shows the distribution and morphology of a sample with high porosity. The calculated porosity level was 3%, which implies the porosity is closed. A shape factor of about 1 is for spherical pores, while an SP of up to 3.36 was obtained for irregular pores. The reasoning behind shape factors implies that the internal stress concentration around the pores is related to the shape factor. However, the average SP is not directly related to the porosity level. It is worth noting that small pores are generally very close to being perfectly round (SP ~ 1), while large pores are irregular and have a significant internal notch effect on mechanical properties.

**Figure 5:**
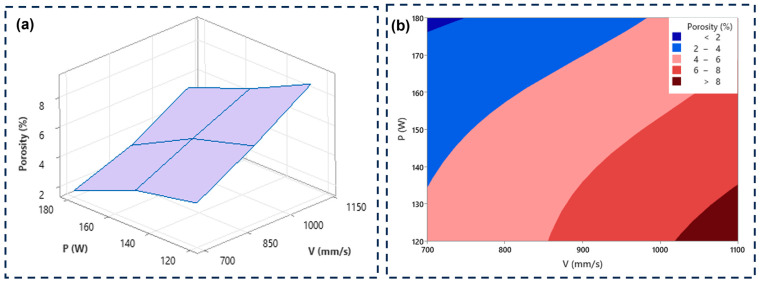
The effect of laser power and scanning speed on the resulting porosity (**a**) surface plot and (**b**) contour plot analysis.

Combining the contour graph and surface plot (Figure 5), a suitable working window can be chosen to control the percentage of porosity by varying laser power and scanning speed. Figure 5a shows a linear behavior, where a higher laser power and a lower scanning speed produces a material with a lower percentage of porosity. Figure 5b shows porosity bands: in the upper left zone is the minimum porosity (<2%); in the central zone, porosity percentages between 2 to 8% can be achieved; and a high porosity is in the lower right zone (>8%).

The analysis of variance of the porosity response is reported in Table 3. As the *p*-Value determines the statistical significance (<0.05), both laser power (P) and scanning speed (V) impacted the porosity percentage, which is in agreement with the findings of the literature [7,8,45].

### 3.2. Tribology Performance

#### 3.2.1. Coefficient of Friction

**Figure 6:**
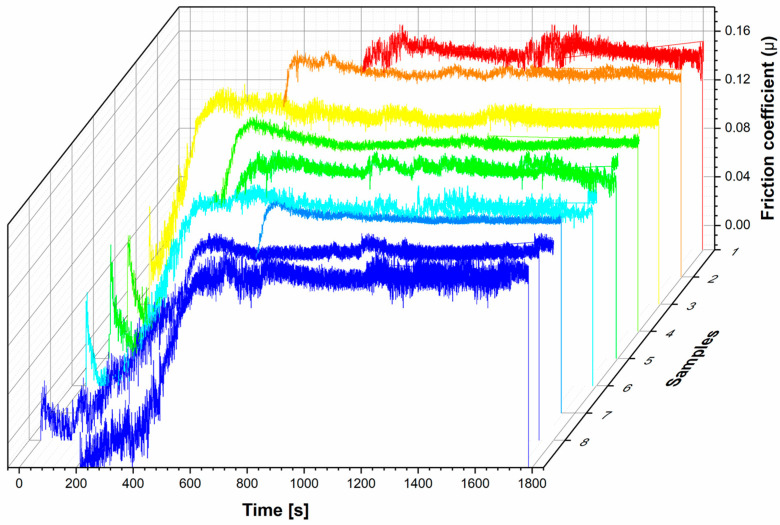
Variation of friction coefficient for the experimental design.

No single model universally describes the synergy between the friction coefficient and wear rate. Several factors, such as surface conditions, hardness, and environmental factors influence this relationship. Figure 6 shows the variation in friction coefficient (µ) during the pin-on-disc test as a function of time. The main difference between the experimental samples is the time to reach the steady-state period. Most samples exceeded the running period of around 400 s (ca. 4 m). In the steady-state period, the µ value varied between 0.122 and 0.151, with an average of 0.137 ± 0.011 (Table 4). However, no relationship between the porosity values and the µ can be observed within the processing parameters evaluated. A comprehensive understanding of the connection between the µ and wear is crucial for enhancing the performance and extending the applicability of biomaterials fabricated by L-PBF.

#### 3.2.2. Wear Resistance

**Figure 7:**
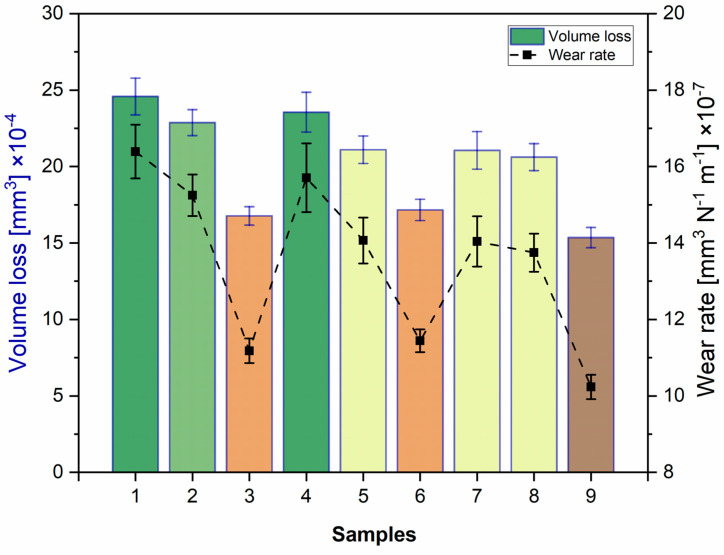
Wear response, evaluation of volume loss, and wear rate.

Figure 7 shows the volume loss and the calculated wear rate of the experimental design after the wear tests. Sample 1 exhibited the maximum material loss (24.56 × 10^−4^ mm^3^), while sample 9 suffered the lowest loss (15.35 × 10^−4^ mm^3^). The wear rate was determined using the Archard relationship [46], and the results showed that it varied slightly between 10 and 17 [mm^3^ N^−1^m^−1^] × 10^−7^ (Table 4). Samples 3, 6, and 9 displayed the lowest wear rate and corresponded to those with the highest porosity level (Table 2).

**Figure 8:**
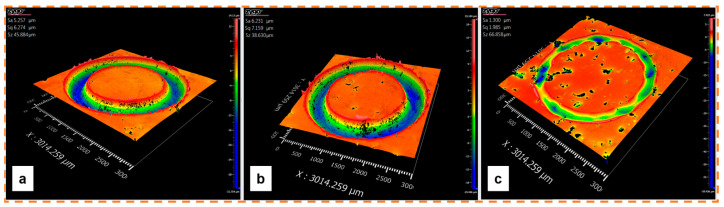
The 3D morphology features of the wear track produced on 316L SS processed with different porosity levels: (**a**) low porosity (S9), (**b**) medium porosity (S6), and (**c**) high porosity (S3). Maximum depths: −31.754, −19.446, and −59.436 µm, respectively.

Figure 8 presents the three-dimensional (3D) surface morphology of the wear track of three representative additively manufactured 316L SS with different porosity levels: low (S9), medium (S6), and high (S3) porosity. Figure 8a exhibited the lowest porosity, with an almost constant wear depth. The wear depth reached a value of around 32 µm. The sample with intermediate porosity showed a reduction in the wear depth (19 µm); nevertheless, it maintained the homogeneity in the wear track. Figure 8c illustrates an irregular wear track where only a few zones exhibited wear marks. This behavior may be associated with the fact that as the material is expelled, the pores serve as a reservoir where the lost material is lodged, and consequently, the wear volume is reduced [16].

#### 3.2.3. Mechanical Properties from Nanoindentation

**Figure 9:**
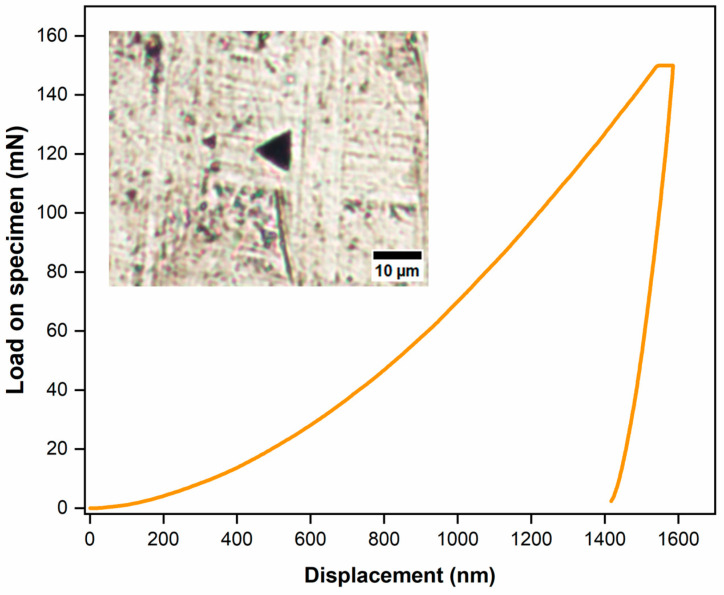
Representative nanoindentation response and OM of a sample with a low porosity level (Berkovich tip indenter).

Figure 9 presents the loading–unloading curve after the nanoindentation test, which is representative for testing biomaterials on the nanometer to micrometer scale. According to Oliver et al. [42,47], nanohardness (*H*) can be used as an indicator of wear resistance. Wear is associated with the capability to withstand severe plastic deformation and can be determined by the ratio between *H*^3^ and *E_r_*^2^. A higher *H*^3^*/E_r_*^2^ relationship reflects a superior wear resistance [48]. Table 5 summarizes the mechanical response of 316L SS on the nanometer scale. Since the material analyzed is the same, the results of *H* and *E_r_* do not significantly differ. Therefore, the *H*^3^*/E_r_*^2^ ratio is almost equal; consequently, no wear response can be obtained.

## 4. Discussion

### 4.1. The L-PBF Process-Induced Porosity

**Figure 10:**
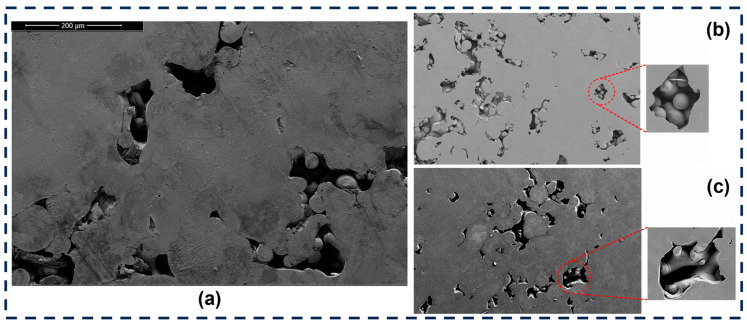
(**a**) Sample with high porosity level; (**b**) porosity due to lack of fusion; and (**c**) porosity with partially melted powder bed.

As seen in Figure 10, the high porosity level is associated with the processing parameters. When insufficient energy is applied to melt the powder bed completely, a lack of fusion occurs where the powder remains intact (Figure 10b). Increasing the volumetric energy density slightly melts the powder bed but does not entirely fuse the raw material. As reported by several authors [13,16,49,50,51], to achieve minimum porosity, an energy density higher than 90 [J mm^−3^] is recommended [45].

**Figure 11:**
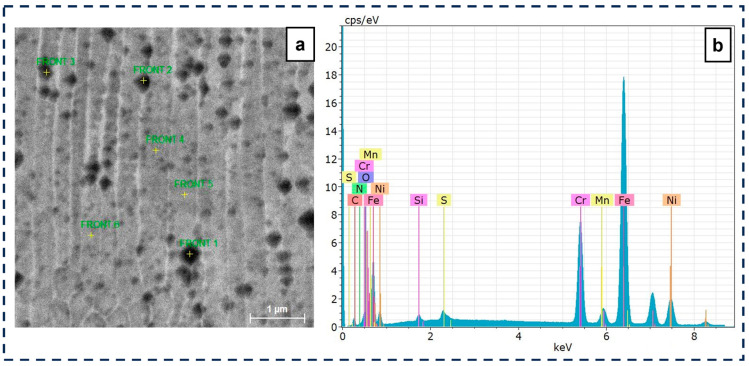
(**a**) SEM micrograph of microporosity and (**b**) EDS analysis of the micropores.

Figure 11 shows the columnar microstructure typical of laser-processed SS, where it is possible to distinguish microporosities [52]. The elemental composition measured on the external surface shows a considerable amount of carbon of around 6%, in addition to the typical values of Fe (66%), Cr (17%), Ni (10.5%), Mo (2.2%), and less than 1% of Si and Mn.

Finding a range of processing parameters is fundamental in L-PBF. As shown in Figure 10 and Figure 11, several porosity types appear during the AM process. While volumetric energy is crucial in avoiding a lack of fusion, microporosities cannot be controlled, as they depend on the raw material [7,23,53,54]. As shown in Figure 8, the level of porosity impacts wear resistance, the effect of which is discussed in the next section.

### 4.2. Porosity Effect on Tribology Properties

According to the experimental design, the dry dynamic coefficient of friction is not influenced by the processing parameters. The µ ranged from 0.122 to 0.151, which agrees with the reported results of conventional manufactured 316L SS [45,46]. A lower friction coefficient generally means less resistance to sliding and less energy dissipation as heat [25]. Thus, the porosity levels obtained for the range of parameters studied do not significantly affect friction.

**Figure 12:**
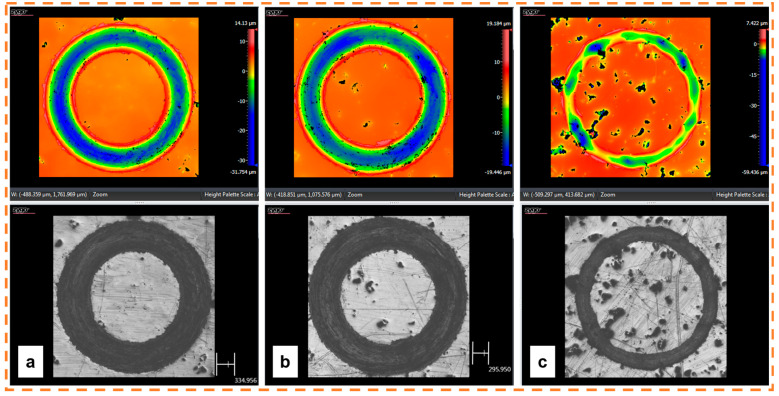
Wear tracks of different porosity levels: (**a**) low porosity (S9), (**b**) medium porosity (S6), and (**c**) high porosity (S3).

Figure 12 shows how the level of porosity impacts the wear track. The sample with lower porosity presents a continuous wear track with an almost homogeneous wear depth. In the sample with intermediate porosity (Figure 12b), it is possible to distinguish areas where the wear track begins to fade due to the appearance of pores that interacted with the pin, thus modifying the wear mechanism. Finally, the wear track is irregular in the specimen with a high porosity level which altered the wear rate (Figure 12c).

**Figure 13:**
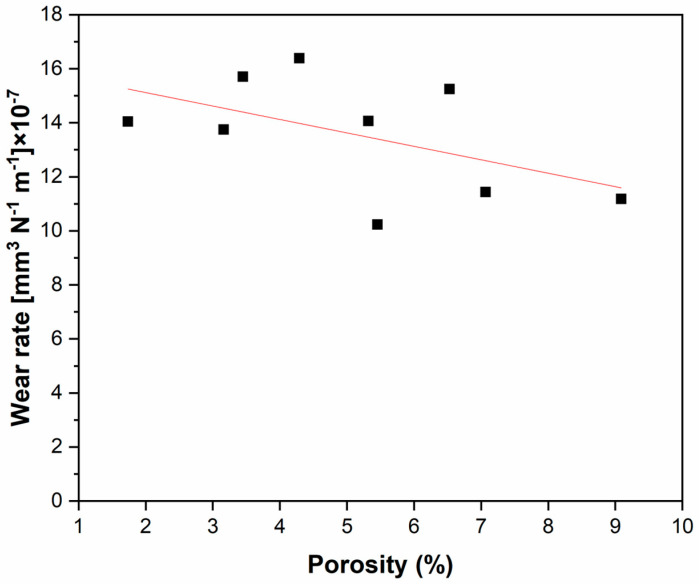
Wear rate as a function of the porosity level of 316L stainless steel manufactured by L-PBF.

Pores play an important role in representing the potential sites of the first microcracks forming and positively influencing the wear process by entrapping the wear debris and preventing the formation of large abrasive agglomerates. Figure 13 shows a trend between porosity level and wear rate. Lower wear losses were obtained when the porosity was higher than 7%.

The overall wear process during sliding is a complex interaction of several sub-mechanisms, e.g., abrasion, adhesion, fatigue, and delamination, each contributing to the overall wear behavior [55]. These sub-mechanisms often operate simultaneously, with their relative contributions depending on factors such as material properties, roughness, sliding speed, loads, and environmental conditions [56]. At a low porosity, adhesive wear prevailed due to the stronger surface interactions and minimal voids. As porosity increased to moderate levels, abrasive wear became dominant as voids weaken the material, allowing micro-cutting and plowing effects. At high porosity, fatigue wear and brittle fracture occurred due to stress concentrations around voids and a reduced structural integrity [2,7,34,36].

In this research, a large number of third bodies (or tribomaterial) were generated during the sliding wear test, of which only a fraction were actually ejected from the tribosystem. The rest may even act as solid lubricants, allowing for smaller rates of material loss. Nevertheless, as abrasive wear progresses, it can expose internal pores, which may then lead to delamination.

**Figure 14:**
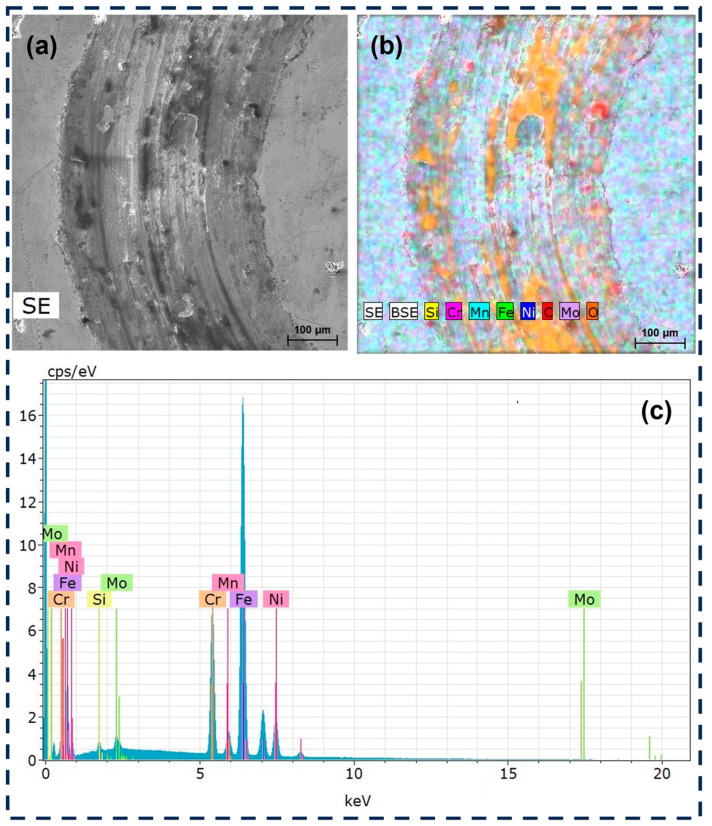
EDS analysis of the wear track: (**a**) SEM image, (**b**) elemental mapping, and (**c**) spectrum of the 316L SS.

EDS analysis of the wear track (Figure 14) points toward mechanical mixing corresponding to a sub-mechanism of tribochemical reactions [40]. Material transfer, deduced from Figure 14b, indicates the adhesion of Si and O, which must have been generated during the rubbing of the deformed 316L SS against the Si_3_N_4_ pin. This would account for mechanical mixing and could protect surfaces if not removed by mechanically dominated mechanisms like abrasion. Figure 14b shows a high concentration of oxygen, indicating an oxidation process, and the oxide layer that formed may be protective. As the oxide layer is brittle, it can fracture and contribute to the wear process by generating abrasive particles.

On the other hand, delamination could also be overfolding. A particular form of microblogging is delamination which is related to surface fatigue, where pores influence crack initialization [57,58]. Nevertheless, subsurface analysis indicated that the primary wear mechanism is overfolding (a phenomenon related to abrasion).

As demonstrated throughout this study, the tribological response can be customized by controlling the porosity level. The most relevant application lies in the biomedical area for prosthesis design, since it would be possible to control the modulus of elasticity and thus reduce stress shielding. However, this behavior must be verified in a lubricated environment before being used in industrial applications.

## 5. Conclusions

The present research explored the effects of porosity resulting from different laser power and scanning speeds in L-PBF-processed 316L stainless steel on tribological performance. Based on microstructural analysis of the wear tracks produced on pin-on-disc testing, it can be concluded that:

- A strong correlation exists between the variables of L-PBF processing and porosity, allowing for the tuning of the latter between 1.7 and 9.1% by selecting a suitable laser power and scanning speed. However, in this continuum of porosity, the choice of a working window should consider the possibility of the lack of fusion for combinations of low/high laser power and scanning speed values.
- There is no evident correlation between porosity and the coefficient of friction; however, materials produced at the highest scanning speed (1100 mm/s) and characterized by higher porosity (>5.5%) tend to be more wear resistant in terms of wear rate, which can be attributed to the capacity of the pores to retain wear debris.
- The primary wear mechanism in dry sliding against a harder material (Si_3_N_4_) is that of three-body abrasion, involving the physical phenomena of adhesion, delamination, oxidation, and tribochemical reactions. Further study of these phenomena is necessary for exploiting the inherent porosity of L-PBF processing to design wear-resistant 316L SS components.
- This study offered a clear advancement in the comprehension of the porosity effect of tribological properties for biomedical prostheses design. However, verifying this behavior in a lubricated environment is necessary before it can be used in industrial applications.

## Figures and Tables

**Table 1:** Processing parameters of the design of experiments.

Factors	Levels		
	−1	0	1
Laser power [W]	120	150	180
Scanning speed [mm/s]	700	900	1100

**Table 2:** Experimental factors and porosity responses. Values of porosity obtained using the Archimedes method and image analysis.

Sample ID	Power [W]	Speed [mm/s]	Archimedes Porosity [%]	Image Analysis Porosity [%]	Average Shape Factor
S1	120	700	4.3 ± 0.5	1.16	1.12
S2	120	900	6.5 ± 0.1	1.21	3.15
S3	120	1100	9.1 ± 1.2	3.02	2.63
S4	150	700	3.4 ± 0.4	1.24	1.28
S5	150	900	5.3 ± 0.4	1.48	1.43
S6	150	1100	7.1 ± 1.4	0.32	1.13
S7	180	700	1.7 ± 0.1	0.21	1.18
S8	180	900	3.2 ± 0.5	0.25	2.09
S9	180	1100	5.4 ± 0.3	0.47	2.31

**Table 3:** Analysis of variance of the porosity percentages.

Source	Df	Adj SS	Adj MS	F-Value	*p*-Value
Model	8	121.39	15.17	21.34	0.00
Linear	4	119.86	29.97	42.15	0.00
P (W)	2	46.03	23.01	32.37	0.00
V (mm/s)	2	73.84	36.92	51.93	0.00
Two-Way Interactions	4	1.53	0.38	0.54	0.71
P × V	4	1.53	0.38	0.54	0.71
Error	18	12.79	0.71		
Total	26	134.19			

Df: degrees of freedom, SS: sum of squares, MS: mean square, F- and *p*-values: statistics.

**Table 4:** Average tribological properties of 316L SS produced by L-PBF.

Sample	Friction Coefficient [-]	Wear Rate [mm^3^ N^−1^ m^−1^] × 10^−7^
S1	0.137 ± 0.018	16.39 ± 0.70
S2	0.146 ± 0.011	15.25 ± 0.54
S3	0.132 ± 0.012	11.18 ± 0.32
S4	0.137 ± 0.009	15.71 ± 0.91
S5	0.129 ± 0.022	14.06 ± 0.66
S6	0.132 ± 0.013	11.44 ± 0.32
S7	0.133 ± 0.010	14.04 ± 0.66
S8	0.138 ± 0.011	13.75 ± 0.51
S9	0.146 ± 0.016	10.23 ± 0.32

**Table 5:** Nanoindentation response of different porosity-level samples processed by L-PBF.

	*H* (GPa)	*E_r_* (GPa)	*H*^3^/*E_r_*^2^
Low-porosity samples	2.84 ± 0.039	141.18 ± 2.48	0.00115
Medium-porosity samples	2.81 ± 0.031	145.22 ± 2.48	0.00105
High-porosity samples	2.76 ± 0.019	142.19 ± 6.24	0.00104

## Data Availability

The original contributions presented in this study are included in the article. Further inquiries can be directed to the corresponding author.
